# Exploring Complexity: A Case Report of a Cystic Hygroma With Complex Congenital Heart Defects and Annular Pancreas in a One-Year-Old Child

**DOI:** 10.7759/cureus.56852

**Published:** 2024-03-24

**Authors:** Chaitanya Kumar Javvaji, Keta Vagha, Sai Bhavani Manchineni, Amar Taksande, Ashish Varma, Punam Uke

**Affiliations:** 1 Pediatrics, Jawaharlal Nehru Medical College, Datta Meghe Institute of Higher Education and Research, Wardha, IND

**Keywords:** paediatric interventional radiology, ventricular septal defect, intralesional bleomycin sclerotherapy, congenital anamolies, cystic hygroma

## Abstract

Lymphatic malformations frequently present as benign masses in the neck and clavicle region among infants and young children. Cystic hygroma represents an often-encountered form of lymphatic malformation. This case report details the medical history of a one-year-old girl characterized by a multifaceted medical background, initially exhibiting symptoms of persistent cough, cold, and neck swelling. Further investigations revealed more severe conditions: complex congenital heart defects, including large atrial septal defect (ASD), large ventricular septal defect (VSD), and aorta arising from the right ventricle with cystic hygroma and annular pancreas. The patient underwent various diagnostic tests, including chest X-rays, ultrasound, magnetic resonance imaging (MRI), and computed tomography pulmonary angiogram (CTPA), leading to multidisciplinary treatment involving sclerotherapy for cystic hygroma and supportive therapies. The case underscores the challenges in diagnosing and managing pediatric patients with overlapping conditions and the critical need for continuous follow-up.

## Introduction

Cystic hygroma is a common type of lymphatic malformation [1]. Cystic hygroma is a benign lesion with a swelling often found in the neck and clavicle region. It develops during embryonic stages due to impaired lymphatic drainage into the venous system [1]. The term "cystic hygroma" was coined by Werner in 1834. On the other hand, the word "hygroma" comes from the Greek word meaning a tumor that contains water, which was first used by Redenbacker in 1828. Despite these early references, the precise origins of these soft tissue tumors remain uncertain [2]. Cystic hygroma occurs in approximately one in every 6,000 live births [3]. Among cystic hygromas, the macrocystic type typically has limited connections with normal lymphatic channels and is more commonly encountered than other lymphangiomas [3]. The onset of cystic hygromas is often noticeable, with more than 60% appearing at birth and nearly 90% becoming clinically evident before age two [4]. These lesions can arise in various anatomical locations, including the cervico-facial regions, mediastinum, axilla, and groin. In addition, they may occur in internal organs, such as the kidney, intestine, spleen, and liver [5,6].

Complications associated with cystic hygromas can vary significantly, often leading to respiratory challenges and swallowing difficulties, particularly when they impact the oral cavity and neck [4]. Although complete surgical removal remains the preferred treatment approach, recent literature increasingly highlights significant success using sclerosant agents [4]. Furthermore, numerous authors have observed the concurrent presence of fetal lymphangioma alongside other congenital malformations [7]. Malone's research, focusing on 65 patients diagnosed with cystic hygroma without chromosomal abnormalities, revealed a notable correlation. In this study, up to 22 cases displayed concurrent cardiac and skeletal defects [8]. We present a unique and complex case involving a one-year-old child diagnosed with cystic hygroma accompanied by complex congenital heart disease and an additional anomaly affecting the pancreas. This case highlights the intricacies of management and underscores the importance of a comprehensive approach in addressing congenital malformations with multiple manifestations.

## Case presentation

**Figure 1:**
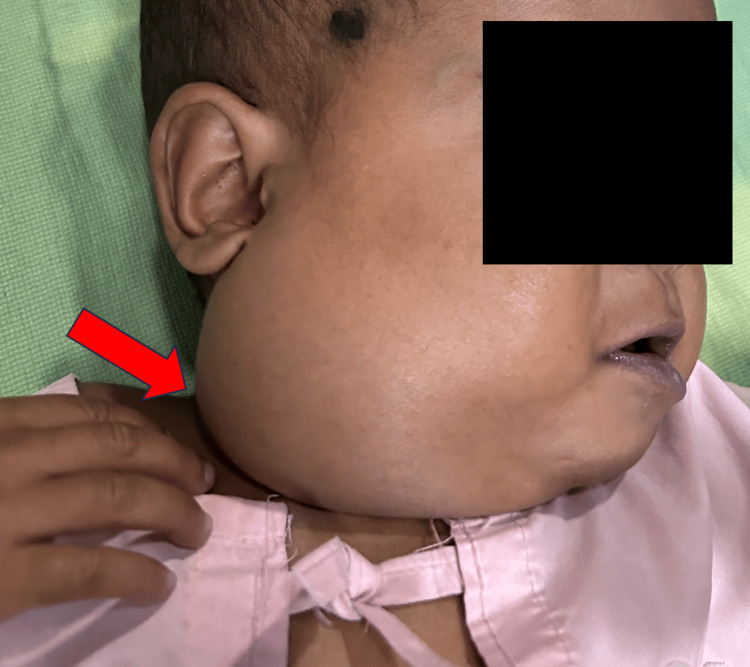
Clinical image depicting a swelling located on the right side of the neck (red arrow)

A one-year-old girl presented to our pediatric outpatient department with a six-month history of recurrent cough and cold, along with swelling on the right side of her neck, persisting for the past three months. According to the father, the child was previously healthy until six months ago when she started experiencing frequent dry cough episodes. In addition, she developed a small swelling on the right side of her neck, which has progressively increased. The swelling is soft, non-tender, and adherent to the skin, with no apparent changes in the overlying skin. The child exhibits a suck-rest-suck cycle and a history of forehead sweating. The parents reported a previous diagnosis of congenital heart disease (CHD), although no medical reports were available. Her antenatal history was uneventful. Upon admission, the child's vital signs were within normal ranges except for an oxygen saturation level of 81%. Upon cardiovascular examination, a pan systolic murmur was detected. A respiratory system examination revealed bilateral crepitations. Central nervous system and abdominal examinations yielded normal findings. Local examination revealed a soft, non-tender swelling on the right side of the neck (Figure 1). Routine investigations revealed elevated total leukocyte count (16,500/cumm), elevated C-reactive protein level (12 mg/dl), and low hemoglobin (7.8 g/dl). The karyotyping analysis revealed a chromosomal composition of 46 XX, indicating a normal female genotype. Other laboratory investigations were within normal limits.

**Figure 2:**
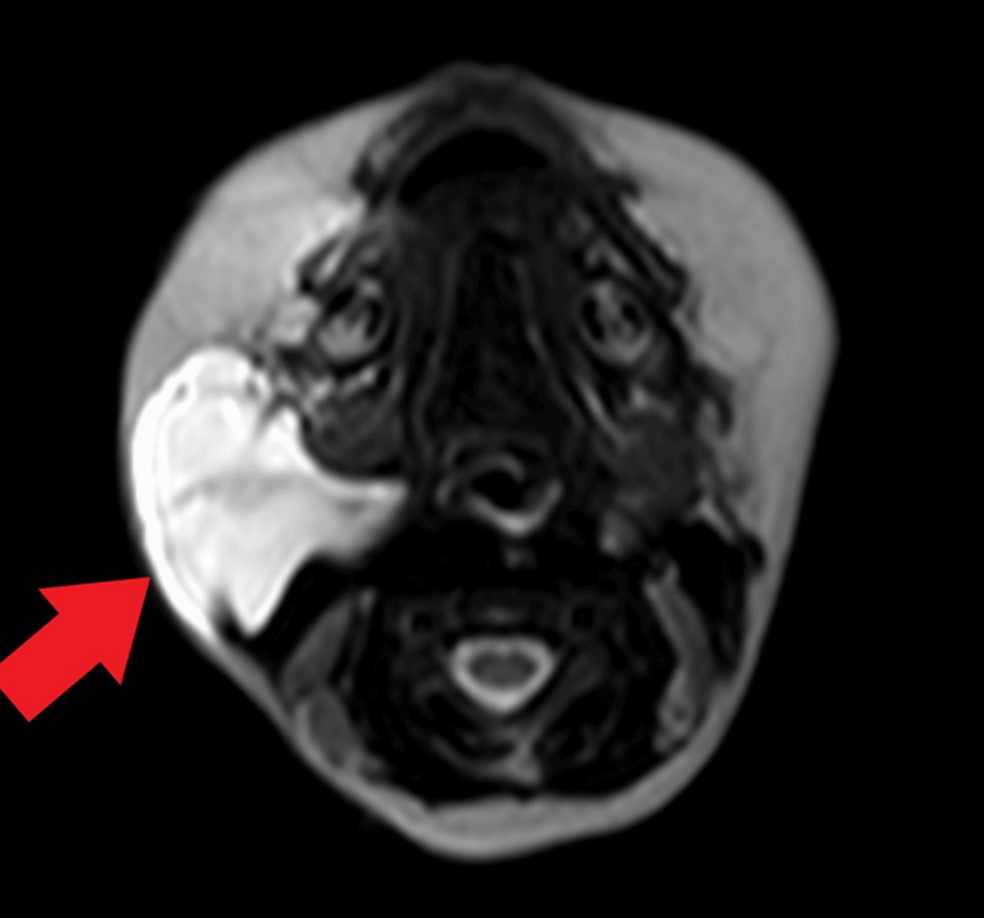
Magnetic resonance imaging revealing cystic lesion in the right submandibular region appearing hyperintense on T2-weighted imaging (red arrow).

Chest X-ray indicated cardiomegaly and consolidation in the upper lobe of the right lung. Ultrasound examination of the swelling revealed a large multiseptated cystic lesion in the posterior cervical space extending to the right parotid region, suggesting cystic hygroma or infective collection in the right parotid gland. The child was initiated on augmentin and nebulization with levosalbutamol. A packed red cell transfusion was administered due to low hemoglobin levels. Pediatric surgery consultation was sought, and a magnetic resonance imaging (MRI) with contrast was recommended to assess the extent and internal structure of the lesion to rule out malignancy. The MRI revealed a cystic lesion in the right submandibular region. It appeared hyperintense on T2-weighted imaging and hypo- to isointense on T1-weighted imaging. There was no evidence of diffusion restriction. The lesion measured approximately 6.5 x 4.3 x 3.1 cm (Figure 2). The lesion was anterior to the right internal carotid artery and sternocleidomastoid muscle, with noted involvement of the parotid space. A review pediatric surgery consultation was conducted, and it was recommended to proceed with sclerotherapy. Following this recommendation, an interventional radiology consultation was arranged, and sclerotherapy was scheduled for the patient.

**Figure 3:**
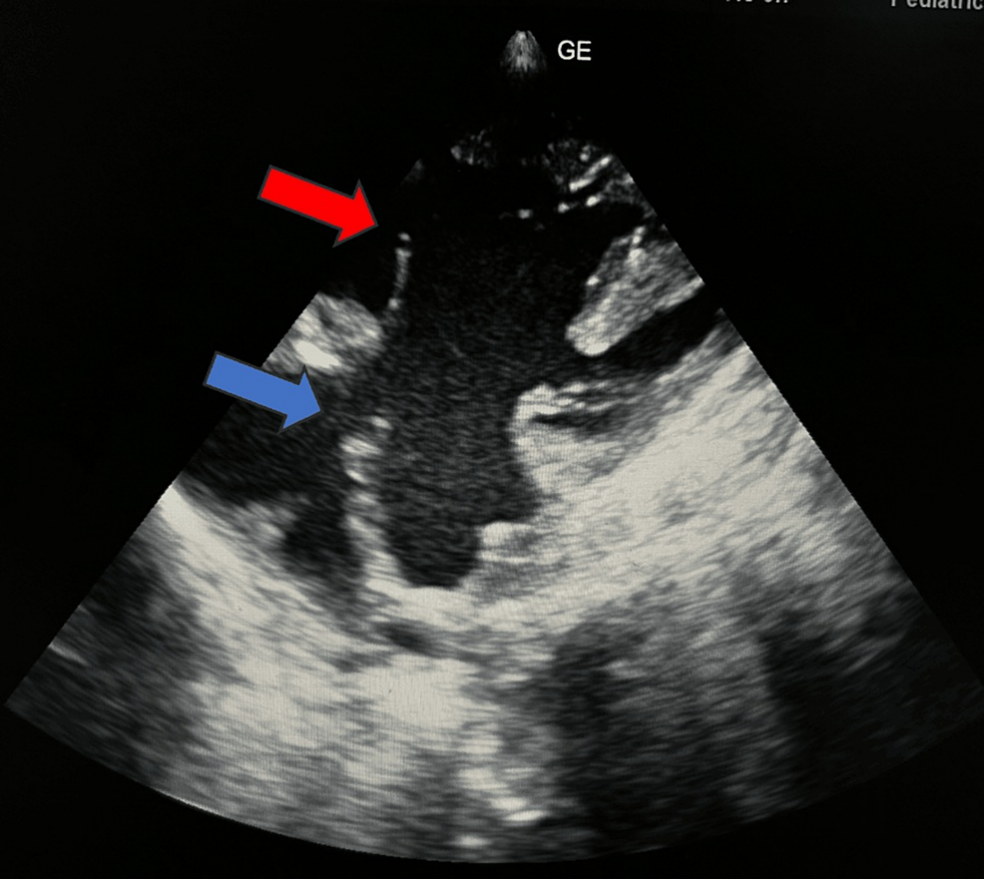
2D echocardiography apical four-chamber view shows large atrial septal defect (blue arrow) and large ventricular septal defect (red arrow).

**Figure 4:**
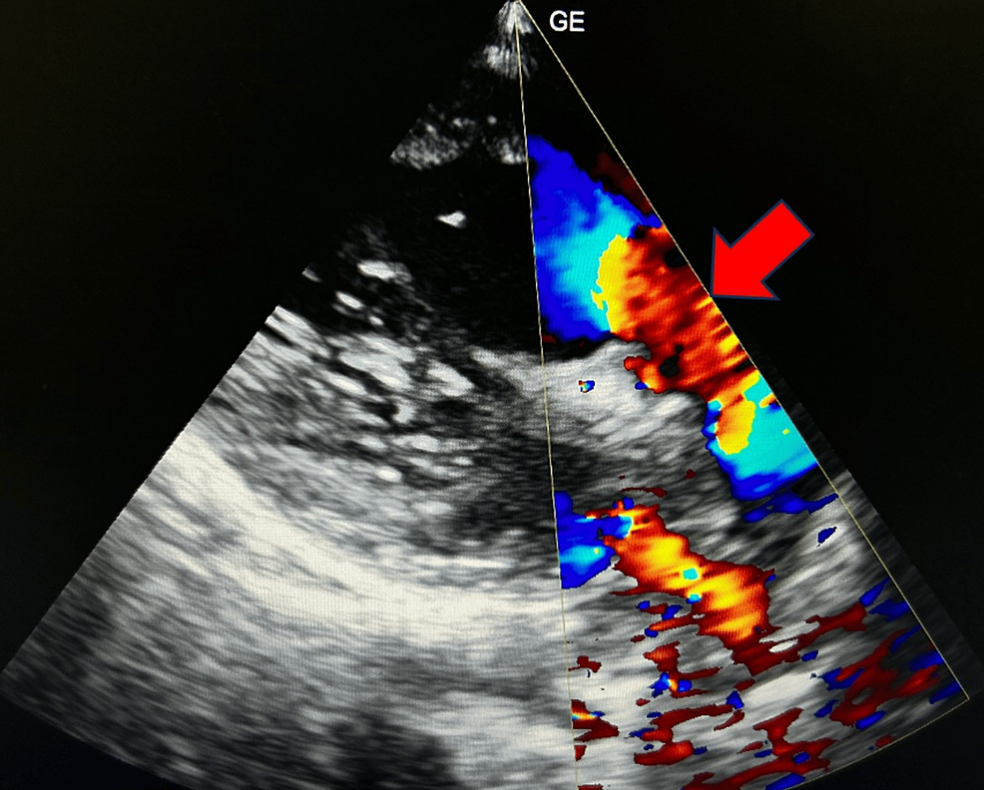
2D echocardiography PLAX view shows aorta arising from the right ventricle (red arrow). PLAX, parasternal long axis

Two-dimensional echocardiography revealed complex CHD with large atrial septal defect (ASD), large ventricular septal defect (VSD), pulmonary stenosis, and aorta arising from the right ventricle (Figure 3, Figure 4).

**Figure 5:**
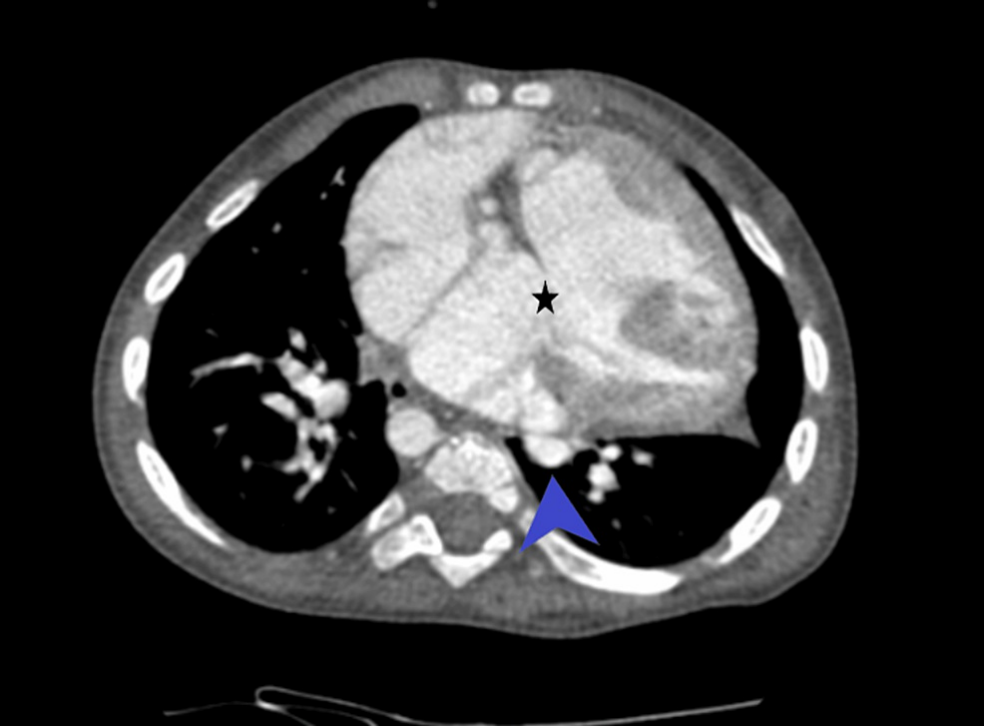
Computed tomography pulmonary angiogram revealing multiple direct and indirect major aortopulmonary collaterals supplying the pulmonary arteries (blue arrowhead), large atrial septal defect, and ventricular septal defect (asterisk).

The computed tomography pulmonary angiogram (CTPA) revealed levocardia accompanied by double-outlet right ventricle (DORV), malpositioning of the great arteries, a right-sided aortic arch, and multiple direct and indirect major aortopulmonary collaterals (MAPCAs) supplying the pulmonary arteries. In addition, there was the presence of a large ASD and VSD (Figure 5).

**Figure 6:**
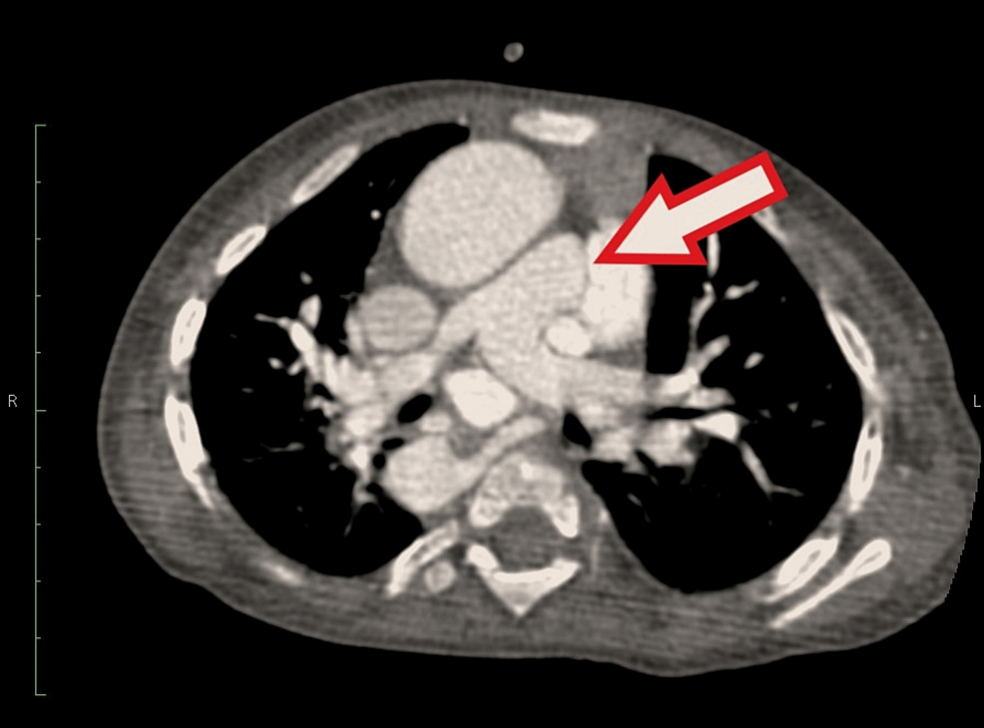
Computed tomography pulmonary angiogram revealing main pulmonary artery and pulmonary valve stenosis (arrow).

The CTPA revealed main pulmonary artery and pulmonary valve stenosis (Figure 6). There was an enlargement of the left pulmonary artery and narrowing of the right pulmonary artery. Consultation with cardiothoracic and vascular surgery was sought, and they recommended a follow-up appointment in two months for cardiac surgery after addressing the treatment of cystic hygroma.

**Figure 7:**
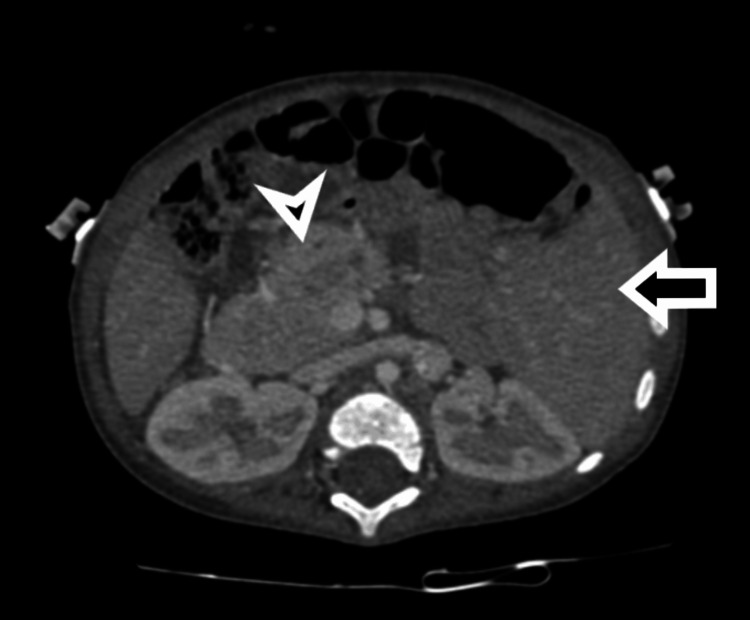
Contrast-enhanced computed tomography imaging of the abdomen revealing a central liver (arrow) with an annular pancreas (arrowhead).

Contrast-enhanced computed tomography (CECT) imaging of the abdomen revealed a central liver with an annular pancreas (Figure 7).

A 24G scalp vein was inserted into the largest pocket of the cystic hygroma under ultrasound guidance. Approximately 10 ml of yellow-colored fluid was aspirated, and the position was confirmed. Subsequently, seven units of bleomycin, along with 2 ml of lipidol and 2 ml of sterol, were injected into the cavity (Video 1). The procedure was uneventful.

**Video 1:** Video demonstrating the injection of sclerosants into the cystic hygroma of the child

The parents received counseling, and the child was discharged with a prescription for cefixime, ibuprofen, lansoprazole, chymoral forte, trypsin-chymotrypsin, and syrup ambroxyl. They were instructed to return for a follow-up appointment in a month for the subsequent cycle of sclerotherapy. However, despite diligent attempts to reconnect, the child was unfortunately lost to follow-up.

## Discussion

Most lymphatic malformations primarily occur in regions with lymphatic vessels, such as the head and neck. However, they can also present in other anatomical areas, including the axillae, mediastinum, groin, and retroperitoneum. Notably, lymphatic malformations affecting the orbit pose a significant concern due to their potential risk to vision [9]. Moreover, giant lymphatic malformations involve critical structures, such as the tongue, base of the oral cavity, cervical region, and mediastinum. These cases often require urgent interventions, such as emergency tracheostomy, staged repair, tube feeding, and long-term speech therapy [1].

During the eighth week of gestation, embryonic development involves the formation of six lymphatic sacs. By the ninth week, connective tissue invasion transforms these sacs into lymph nodes. Cystic hygromas are thought to embryologically originate from the sequestration of lymphatic tissue during the development of lymphatic-venous sacs. These sequestered tissues fail to connect with the remainder of the lymphatic or venous system, leading to subsequent dilation and the distinctive cystic morphology observed in these lesions [10].

Cystic hygroma often occurs alongside other malformation syndromes and is frequently associated with additional anomalies. Turner syndrome is the most common abnormality found in approximately 50% to 70% of cases of cystic hygroma. Fetuses affected by cystic hygroma and Turner syndrome commonly exhibit concurrent abnormalities in cardiac development, including defects in the aortic valve, hypoplasia of the third part of the aortic arch, anomalous origin of the branches of the aortic arch, and septal defects [11]. The exact pathogenesis of lymphatic system malformations remains incompletely elucidated. Two hypotheses regarding the development of lymphatic malformations have been posited: one suggests abnormalities or inadequacies within the lymphatic system network. At the same time, the other proposes deficiencies in the connection between the lymphatic and venous systems [12]. The latter hypothesis is considered more plausible for cystic hygromas occurring in the neck and head, given the shared developmental stages and connections between the two vascular systems during embryonic development [12].

Ultrasound imaging typically reveals a multicystic lesion with internal septations, and color Doppler ultrasonography shows no detectable blood flow. Other modalities, such as CT scans and MRI, are often utilized for more detailed lesion visualization. The prenatal detection of cystic hygroma via ultrasound is extensively documented, often with malformation in the nuchal region. The typical sonographic presentation on antenatal ultrasonography usually manifests as a multiseptate, thin-walled cystic mass, occasionally displaying a more intricate echo texture comprising solid and cystic components [4].

In approximately 62% of cases, a fetus with cystic hygroma may present with associated anomalies, such as Noonan syndrome, Down's syndrome, Turner's syndrome, Trisomy 13, and Trisomy 18 [4,13]. Although cystic hygromas are benign lesions, complications can arise, including infection and spontaneous bleeding within the cyst. Infections typically originate from secondary foci, such as respiratory tract infections, although primary infections can also occur. Spontaneous rupture of giant cystic hygromas, necessitating urgent surgical intervention, has been reported in the literature [5].

Differential diagnoses for cystic hygromas include thyroglossal cyst, laryngocele, dermoid cyst, thymic cyst, brachial cleft cyst, thyroid mass, lipoma, and hemangioma. Most experts recommend MRI for accurate diagnosis and surgical planning [14]. Traditionally, surgical intervention has been the standard therapeutic approach for these lesions. However, due to high recurrence rates and technical challenges associated with resection, particularly given the proximity to vital structures and poorly demarcated lesion margins, there has been a growing interest in exploring alternative treatment methods [1]. Currently, several alternative treatment options are available, including conservative approaches, such as injecting sclerosing agents, repeated content aspiration, radiotherapy, and radiofrequency ablation [12]. 

The concept of using sclerotherapy for lymphangioma treatment arose from observations of spontaneous involution following infection resolution. The initial recorded instance of sclerotherapy for cystic hygroma dates back to 1933, employing sodium morrhuate as the sclerosant agent [15]. Historically, sclerosant agents, such as nitromin, doxycycline, urethane, boiling water, and quinine, were utilized for sclerotherapy. However, the utilization of these agents was linked to frequent complications and low success rates [16]. Available sclerosing agents include iodine, OK-432 (Picibanil), bleomycin, ethanolamine oleate, cyclophosphamide, ethic, tetracycline, and absolute alcohol. OK-432 and bleomycin are two of the most frequently employed sclerosants for sclerotherapy in cystic hygromas [16].

In 1965, Japanese scientist Dr. Hamao's pioneering work led to bleomycin's development. This pharmacological agent operates by inducing fragmentation in both double-stranded and single-stranded deoxyribonucleic acid (DNA), effectively inhibiting the synthesis of deoxyribonucleic acid (DNA) and ribonucleic acid (RNA). Originally designed to address malignant pleural effusion, bleomycin's sclerosing properties were initially applied in 1977 when Yura utilized it to treat cystic hygroma [16]. Adverse effects commonly associated with bleomycin therapy include transient swelling, fever, hematoma formation, hemorrhage, and pulmonary fibrosis. However, a study conducted by Anoop et al. reported no significant complications resulting from intralesional bleomycin sclerotherapy [17].

Alternatively, OK-432 can be utilized as a sclerotherapy agent. Sclerotherapy with OK-432 for lymphatic malformations generally results in excellent clinical responses in most patients. This approach is deemed safe, with minimal occurrence of severe side effects. Common post-injection symptoms include redness at the injection site, swelling, pain, and low-grade fever, although these symptoms do not necessarily correlate with treatment outcomes [2].

In our case, following consultations with pediatric surgery, cardiothoracic vascular surgery, and interventional radiology, a collective decision was made to pursue sclerotherapy due to its minimally invasive nature and reduced risk of complications [17]. Therefore, intralesional bleomycin sclerotherapy was chosen as an alternative to surgical excision for treating cystic hygroma.

## Conclusions

Although cystic hygroma is frequently observed in pediatric patients, it can occur at any age, necessitating its inclusion in the range of potential differential diagnoses across all age groups. Sclerotherapy encounters fewer complications in contrast to complete surgical excision. This case report of a one-year-old with cystic hygroma, complex heart defects, and an annular pancreas emphasizes the challenges in pediatric healthcare. Mainly, it showcases the intricacies of diagnosing and treating cystic hygroma, a lymphatic malformation common in children, in the presence of other severe conditions. The successful management of sclerotherapy for cystic hygroma and treatments for cardiac issues underlines the importance of a multidisciplinary approach and continuous follow-up in complex pediatric cases. This case contributes valuable insights into handling rare and intricate conditions in pediatric patients.
